# Pericardial-Peritoneal Window as an Alternative Treatment for Large and Recurrent Pericardial Effusion Post-Pericardiotomy

**DOI:** 10.21470/1678-9741-2020-0179

**Published:** 2022

**Authors:** Yongbo Kang, Yue Cai, Wei Pan

**Affiliations:** 1 School of Basic Medical Sciences, Shanxi Medical University, Taiyuan, Shanxi, China.; 2 Medical Faculty, Kunming University of Science and Technology, Kunming, Yunnan, China.; 3 Aerospace Breeding Research Centre of CASTC, Beijing, China.

**Keywords:** Foam Cells. Drug-Eluting Stents. Sirolimus. Paclitaxel, Cytokines. Coronary Restenosis. Transforming Growth Factor beta. Anti-Inflammatory Agents. Percutaneous Coronary Intervention. Myocytes, Smooth Muscle

## Abstract

**Introduction:**

Drug-eluting stents (DES) coated with rapamycin or paclitaxel as antiproliferative substances significantly reduced the incidence of clinical restenosis and had fewer side effects after percutaneous coronary intervention. However, DES coated with rapamycin or paclitaxel still cause restenosis due to abnormal tissue growth which remained a therapeutic problem, particularly in certain subgroups, possibly due to drug concentrations. This study examined the impact of different concentrations of rapamycin and paclitaxel on cytokine, cell viability and proliferation in human aortic smooth muscle cells (HASMC)-derived foam cells.

**Methods:**

The foam cell model was established *in vitro* by incubating HASMC with 20 µg/mL oxidized low-density lipoprotein (ox-LDL) for 48 hours. Subsequently, foam cells were treated with different concentrations (0.01 µg/mL, 0.1 µg/mL, 0.5 µg/mL, 1 µg/mL, 5 µg/mL and 10 µg/mL) of rapamycin or paclitaxel for 48 hours, to measure cytokine, cell viability and proliferation by ELISA and MTT, respectively. Finally, viability and proliferation were measured by MTT after the foam cells were treated with 1 µg/mL rapamycin or paclitaxel combined with cytokine antibody for 48 hours.

**Results:**

After incubation of HASMC with ox-LDL, the ratios of cholesterol ester and total cholesterol increased significantly (55.29%) (*P*<0.01). Lipid staining with Oil Red O showed many lipid vacuoles and red dye particles in the cells. Meanwhile, cell viability and proliferation significantly increased compared with the control. This indicated that HASMC had been transformed into foam cells (*P*<0.01) while rapamycin or paclitaxel concentrations ≥0.1 µg/mL can significantly decrease the foam cell proliferation (*P*<0.05 or *P*<0.01), and 1 µg/mL of rapamycin or paclitaxel appeared the most effective concentration. As for cytokines, rapamycin or paclitaxel concentrations ≥1 ug/mL could significantly increase the level of inflammatory cytokines IL-6 (*P*<0.05 or *P*<0.01), which was enhanced with the increase of drug concentration. However, rapamycin or paclitaxel concentrations ≥1 µg/mL could significantly reduce the levels of anti-inflammatory cytokines IL-35 and transforming growth factor beta (TGF-β) (*P*<0.05 or *P*<0.01), which decreased with the increase of drug concentration. In addition, rapamycin or paclitaxel combined with anti-IL-1β, anti-IL-6, anti- TNF-α or anti-IL-35 had no significant effect on foam cell proliferation compared to the drug alone. However, rapamycin or paclitaxel combined with anti-IL-10 or anti-TGF-β can significantly enhance foam cell proliferation (*P*<0.01). In addition, there was no difference in the effects of the same concentrations of rapamycin and paclitaxel on foam cells.

**Conclusion:**

Although rapamycin or paclitaxel can reduce foam cell proliferation, too high or too low concentrations could decrease effectiveness. In particular, a high dose can induce foam cells to increase inflammatory cytokines secretion, reduce anti-inflammatory cytokines secretion, and thus affect the inhibiting proliferation. For rapamycin- and paclitaxel-eluting stents, this conclusion may explain the clinical observation of in-stent restenosis after percutaneous coronary intervention. DES coated with an appropriate concentration of rapamycin or paclitaxel may, at least to some extent, contribute significantly to reducing incidence of late in-stent restenosis.

**Table:** 

Abbreviations, acronyms & symbols				
ANOVA	= Analysis of variance		mTOR	= Mammalian target of rapamycin
ATCC	= American Tissue Culture Collection		Ox-LDL	= Oxidized low-density lipoprotein
DES	= Drug-eluting stents		PBS	= Phosphate-buffered saline
ELISA	= Enzyme-linked immunosorbent assay		SMC	= Smooth muscle cells
FBS	= Fetal bovine serum		SPSS	= Statistical Package for the Social Sciences
HASMC	= Human aortic smooth muscle cells		TGF-β	= Transforming growth factor beta

## INTRODUCTION

Intimal hyperplasia after stent placement and the resultant restenosis remain problematic despite numerous improvements in stent technology and placement technique ^[1-5]^. In the last decade, drug-eluting stents (DES) coated with antiproliferative agents have been the focus of considerable research due to their potential to eliminate restenosis ^[6,7]^. DES coated with rapamycin or paclitaxel as antiproliferative substances significantly reduced the incidence of clinical restenosis and had fewer side effects ^[8,9]^. However, DES coated with rapamycin or paclitaxel may lead to restenosis due to abnormal tissue growth, which remains a therapeutic problem, particularly in certain subgroups, possibly due to insufficient local drug concentrations ^[10-12]^. In general, DES contains a fixed amount of rapamycin or paclitaxel per unit of metal surface area (about 140 mg per cm^2^) ^[13,14]^. Meanwhile, after treated with DES coated with rapamycin or paclitaxel, the in-stent restenosis rate is 37% and 21%, respectively ^[3,15,16]^.

Rapamycin inhibits smooth muscle cell (SMC) proliferation and migration by inhibiting the mammalian target of rapamycin (mTOR). This will lead to a cell cycle arrest at the point from G_1_ to the S phase ^[17]^. Paclitaxel reduces the availability of tubulin, essential for mitosis, via stabilizing microtubules ^[18]^, thereby preventing migration and causing a cell cycle arrest at the G_O_/G_1_ or G_2_/M transition ^[18,19]^. Delivering medication directly to the vascular injury site via polymer-coated stents seems a rational approach to achieve adequate local drug delivery. In addition, the increased risk of late stent thrombosis is a major concern after DES implantation ^[20-23]^.

In this study, we determined the effects of different concentrations of rapamycin and paclitaxel on inflammatory cytokine, cell viability and proliferation in human aortic smooth muscle cells (HASMC)-derived foam cells. To further evaluate such effectiveness, an additional study of activation and proliferation of foam cells via rapamycin or paclitaxel combined with cytokine antibody was conducted.

## METHODS

### Culture of HASMC

The HASMC line purchased from China Shanghai 3Bio Biotechnology Co., Ltd was cultured in DMEM/F12 medium (Gibco, Grand Island, USA) supplemented with 10% fetal bovine serum (FBS) (Gibco, Grand Island, USA) and 1% penicillin-streptomycin (Gibco, Grand Island, USA). Cells were maintained in 5% CO_2_ at 37 ^o^C in a humidified incubator.

Preparation and Culture of HASMC-Derived Foam Cells 

Human aortic smooth muscle cells line in T25 flask were washed and dissociated, and subsequently placed into a 6-well plate (6×10^6^ cells/well). When cell proliferation was at approximately 50%, the culture medium was discarded, and the cell monolayers were washed twice with phosphate-buffered saline (PBS). Then, the cells were added with 3 mL of culture medium containing 20 µg/mL of human lipoprotein oxidized-low density (ox-LDL), and then incubated at 37 ^o^C with 5% CO_2_ for 48 hours. Culture medium without ox-LDL was used as a control.

### Staining of HASMC-Derived Foam Cells

When HASMC growth was at about 80%, they were seeded in coverslips in 6-well plates, and then incubated for 48 hours in 2 mL DMEM/F12 medium containing 20 µg/mL of ox-LDL. Thereafter, the media were aspirated, and the cells were rinsed in PBS followed by formalin fixation for 10 minutes. Then, the cells were rinsed once again with PBS (1 min) and 60% isopropanol (15 s). This was followed by staining of cells with Oil Red O solution 0.5% for 15 minutes at room temperature; cells were destained with 60% isopropanol for 15 seconds followed by PBS washing. Nuclei were stained with Harris hematoxylin and coverslips were mounted on glycerin jelly. Images were obtained using an Olympus BX40 microscope at 4× magnification.

### Quantification of Cholesterol Uptake of HASMC-Derived Foam Cells

Total cholesterol and free cholesterol content were analyzed using the Total Cholesterol Assay Kit and the Free Cholesterol Assay Kit (Applygen, Beijing, China), according to the manufacturer’s instructions. Total cholesterol consists of free cholesterol and cholesterol ester, so cholesterol ester content is equal to the total cholesterol content minus the free cholesterol content.

### Proliferation of HASMC-Derived Foam Cells

Primary HASMC cultures were plated (8×10^4^ cells/well) in 96-well tissue culture plates in 2 mL DMEM/F12 medium containing 20 µg/mL of ox-LDL. After incubation for 48 hours, 20 µL of 0.5 mg/L MTT solution were added to each well, and it was maintained in 5% CO_2_ at 37 ^o^C for 4 hours in a humidified incubator. Thereafter, the culture supernatant was removed, 150 µL of DMSO were added to each well, and it was shaken in a shaker at 37 ^o^C for 10 minutes. The OD value of each well was measured at 490 nm after the crystallization was dissolved.

### Treatment of HASMC-Derived Foam Cells with Rapamycin or Paclitaxel

HASMC-derived foam cells were treated with different concentrations (0.01 µg/mL, 0.1 µg/mL, 0.5 µg/mL, 1 µg/mL, 5 µg/mL and 10 µg/mL) of rapamycin or paclitaxel for 48 hours for cytokine and proliferation analysis.

### Quantification of Cytokines

After the media were collected and centrifuged, the released levels of cytokines (IL-1β, IL-6, TNF-α, IL-10, IL-35 and TGF-β) were determined with commercial ELISA kits (Applygen, Beijing, China), according to the manufacturer’s protocols. In general, cytokine antibody-coated plates were cultured with 5-fold dilutions of sample at 37 ^o^C for 30 minutes. The plates were washed and then incubated for 30 minutes with horseradish peroxidase-conjugated cytokine antibody. The plates were then washed, treated with tetramethylbenzidine, and incubated for 15 minutes. Finally, sulfuric acid was added to end the reaction. The absorbance was then examined at 450 nm UV with a microplate reader (Potenov, Beijing, China) and the inflammatory cytokine concentration was calculated based on the standard curve.

### Treatment of HASMC-Derived Foam Cells with Rapamycin or Paclitaxel Combined with Cytokine Antibody

HASMC-derived foam cells were treated with 1 µg/mL of rapamycin or paclitaxel combined with anti-IL-1β (1:500), anti-IL-6 (1:2500), anti-TNF-α (1:500), anti-IL-10 (1:50000), anti-IL-35 (1:300) or anti-TGF-β (1:300) for 48 hours for proliferation analysis (Abcam, Cambridge, UK).

### Statistical Analysis

A statistical comparison of the data was carried out using the two-way analysis of variance (two-way ANOVA) and *P*<0.05 was considered significant. The results were expressed as mean±SD. The analysis was performed with the SPSS software.

## RESULTS

### Foam Cell Transformation of Smooth Muscle Cells Induced by Ox-LDL

**Fig. 1:**
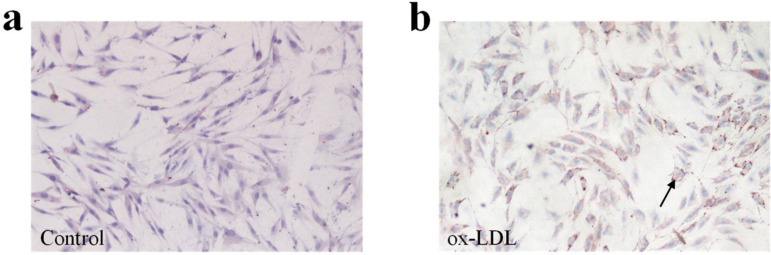
Foam cell transformation of HASMC after incubated with 20 ug/mL of ox-LDL as shown by staining with Oil Red O at 4× magnification. a: control; b: ox-LDL. The lipid vacuoles are indicated by arrows.

After incubation of human smooth muscle cells (SMC) with 20 µg/mL of ox-LDL for 48 hours, lipid staining with Oil Red O became positive, meanwhile there were many lipid vacuoles and many red lipid drops in the cells (Figure 1B). This indicates that the SMC of the aorta have been transformed into foam cells. Detectable foam cell transformation did not occur in the control (Figure 1A). 

**Fig. 2:**
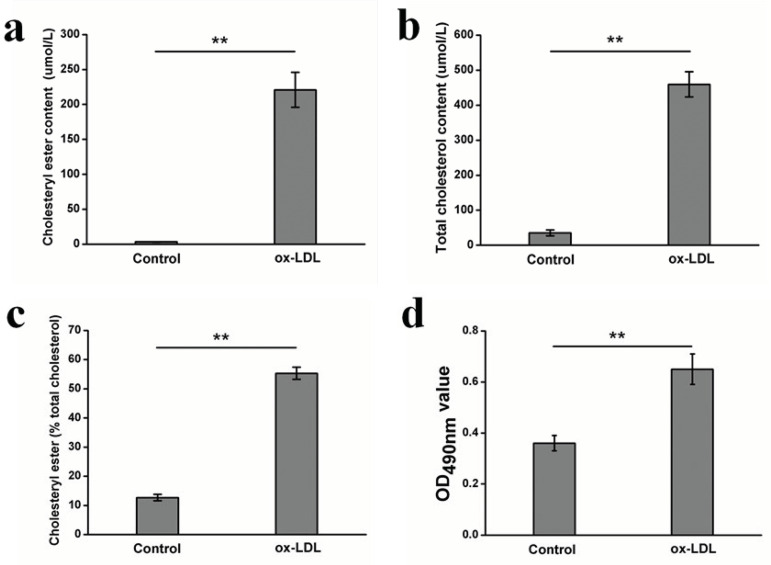
Effects of ox-LDL on HASMC. a: cholesterol ester content; b: total cholesterol content; c: ratio of cholesterol ester to total cholesterol; d: proliferation.

Measuring the amount of cholesteryl ester and total cholesterol confirmed the cellular accumulation of sterol, which increased significantly after 48 hours of incubation with ox-LDL, compared with the control (Figure 2A and B) (*P*<0.01). Meanwhile, the ratios of cholesterol ester and total cholesterol also increased significantly in the ox-LDL-induced human SMC (55.29%) compared with the control (12.70%) (Figure 2C) (*P*<0.01).

**P<0.01 ox-LDL versus control.

In addition, the effects of ox-LDL on SMC proliferation were examined after 48 hours of co-culturing. Compared with the control, ox-LDL significantly increased SMC proliferation (Figure 2D) (*P*<0.01).

In conclusion, all these results suggested that foam cell transformation of SMC was induced after 48 hours of incubation with 20 µg/mL of ox-LDL.

### Treatment of HASMC-Derived Foam Cells with Rapamycin or Paclitaxel

To assess the impact of different concentrations of rapamycin or paclitaxel administration on HASMC-derived foam cells, inflammatory and anti-inflammatory cytokines were first analyzed by ELISA after 48 hours after treatment. 

**Fig. 3:**
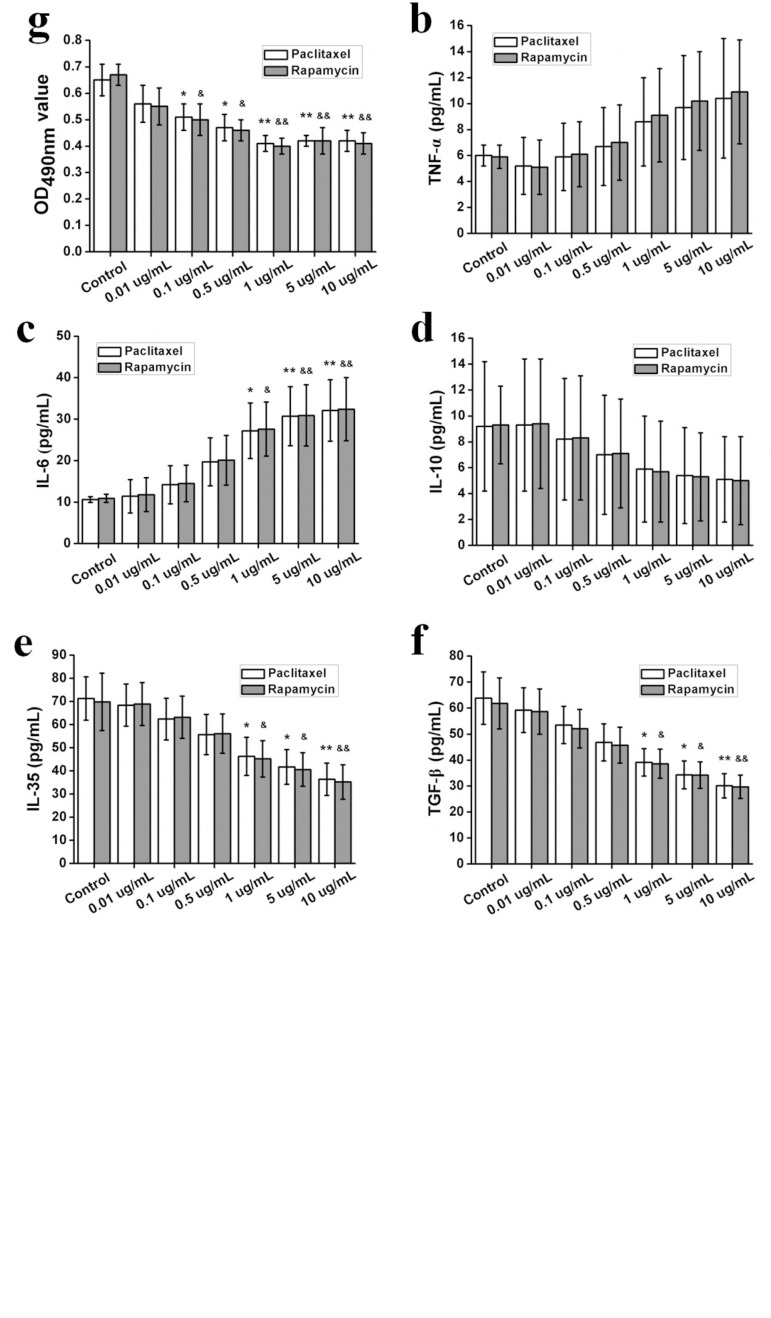
Effects of different concentrations of rapamycin or paclitaxel administration on HASMC-derived foam cells. a: IL-1β; b: TNF-α; c: IL-6; d: IL-10; e: IL-35; f: TGF-β; g: proliferation.

As for inflammatory cytokines, the treatment of HASMC-derived foam cells with rapamycin or paclitaxel had no significant effect on IL-1β and TNF-α levels (Figure 3A and B), whereas the treatment significantly increased the IL-6 level at concentrations of 1 ug/mL, 5 ug/mL and 10 ug/mL (Figure 3C) (*P*<0.05 or *P*<0.01). 

*P<0.05 and **P<0.01 paclitaxel versus control; &P<0.05 &&P<0.01 rapamycin versus control.

As for anti-inflammatory cytokines, the treatment of HASMC-derived foam cells with rapamycin or paclitaxel had no significant effect on the levels of IL-10 (Figure 3D), while it significantly decreased IL-35 and the levels of transforming growth factor beta (TGF-β) at concentrations of 1 ug/mL, 5 ug/mL and 10 ug/mL (Figure 3E and F) (*P*<0.05 or *P*<0.01). To determine the influence of rapamycin and paclitaxel on the foam cell proliferation, MTT experiments were performed. Proliferation was found to be significantly reduced after 48 hours after treatment of SMC with rapamycin or paclitaxel at concentrations of 0.1 ug/mL, 0.5 ug/mL, 1 ug/mL, 5 ug/mL and 10 ug/mL, compared to untreated cells (Figure 3G) (*P*<0.05 or *P*<0.01). Meanwhile, the lowest proliferation rate was observed in the treatment of SMC with rapamycin or paclitaxel at a concentration of 1 ug/mL (Figure 3G).

### Effect of Treatment with Rapamycin or Paclitaxel Combined with Cytokine Antibody on Foam Cell Proliferation

**Fig. 4:**
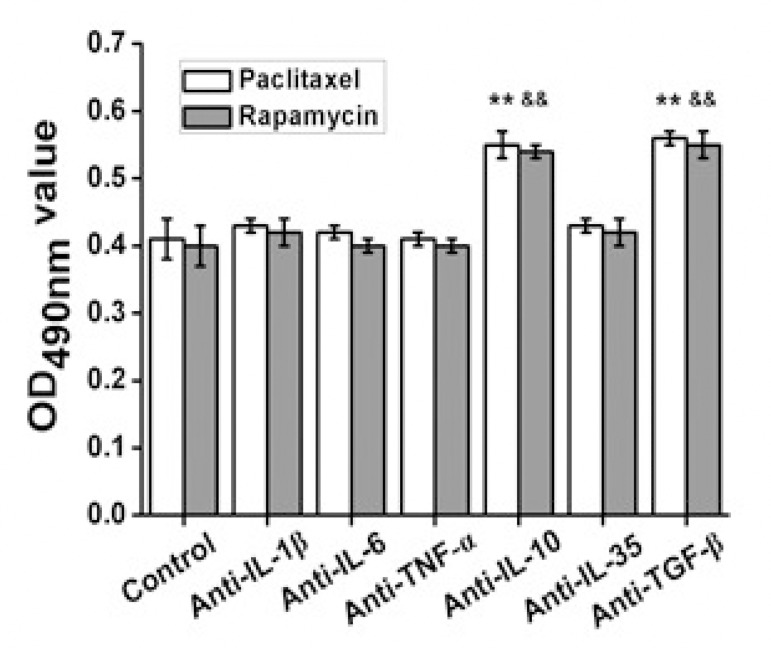
Effect of treatment with rapamycin or paclitaxel combined with cytokine antibody on foam cells proliferation.

To demonstrate the hypothesis that rapamycin or paclitaxel affected the foam cell proliferation by affecting cytokines, the foam cells were treated with rapamycin or paclitaxel combined with cytokine antibody for 48 hours. The proliferation showed no significant difference in the foam cells treated with rapamycin or paclitaxel combined with anti-IL-1β, anti-IL-6, anti-TNF-α or anti-IL-35 compared with the control (Figure 4). However, the proliferation was significantly enhanced in foam cells treated with rapamycin or paclitaxel combined with anti-IL-10 or anti-TGF-β (Figure 4) (*P*<0.01).

**P<0.01 paclitaxel versus control; &&P<0.01 rapamycin versus control.

## DISCUSSION

The HASMC-derived foam cell proliferation plays a key role in the pathogenesis of atherosclerosis. A large number of studies suggested that the insudation of LDL in HASMC can invoke foam cell transformation ^[24,25]^. Meanwhile, ox-LDL is known to accumulate in HASMC to transform foam cells *in vitro* ^[26-28]^. In our study, foam cell model was established *in vitro* by incubating HASMC with 20 µg/mL of ox-LDL for 48 hours. 

To study the effect of rapamycin or paclitaxel on foam cell proliferation, the foam cells were subsequently treated with different concentrations (0.01-10 ug/mL) of rapamycin or paclitaxel for 48 hours. It was found that rapamycin or paclitaxel could reduce foam cell proliferation. 

Although concentrations of rapamycin or paclitaxel ≥0.1 µg/mL could significantly decrease foam cell proliferation, the proliferation was the lowest after treatment of foam cells with 1 ug/mL of rapamycin or paclitaxel. Some studies demonstrated that DES coated with rapamycin or paclitaxel could still cause in-stent restenosis after percutaneous coronary intervention ^[8,9,29,30]^. Meanwhile, our study supported the hypothesis that such restenosis was induced by insufficient local drug concentrations, which was also speculated by other researchers ^[29,30]^. In addition, we found that drug concentrations ≥1 µg/mL resulted in decreased therapeutic efficacy. 

Some studies showed that cytokines secreted by foam cells can induce abnormal proliferation ^[31-34]^. Thus, our study also investigated the effect of rapamycin or paclitaxel on cytokine secretion of foam cells. Rapamycin or paclitaxel concentrations ≥1 µg/mL could significantly increase the level of inflammatory cytokines IL-6, meanwhile the higher the drug concentration, the higher the level of IL-6. However, rapamycin or paclitaxel concentrations ≥1 µg/mL can significantly reduce the levels of anti-inflammatory cytokines IL-35 and TGF-β, and the higher the drug concentration, the lower the levels of IL-35 and TGF-β. Considering the above results, it was speculated that the drug in high doses could induce foam cells to secrete cytokines, which thereby decreases the effect of inhibiting proliferation.

To verify the validity of the hypothesis, the study investigated the effect of treatment with rapamycin or paclitaxel combined with cytokine antibody on foam cell proliferation. It was found that the proliferation was significantly enhanced in the foam cells treated with rapamycin or paclitaxel combined with anti-IL-10 or anti-TGF-β, which demonstrated that the hypothesis was correct.

As for rapamycin and paclitaxel-eluting stents, this may explain the clinical observation of in-stent restenosis after percutaneous coronary intervention ^[35]^.

## CONCLUSION

DES coated with an appropriate concentration of rapamycin or paclitaxel may, at least to some extent, contribute significantly to reducing the incidence of late in-stent restenosis.

**Table:** 

Authors' roles & responsibilities	
YK	Substantial contributions to the conception or design of the work; or the acquisition, analysis or interpretation of data for the work; drafting the work or revising it critically for important intellectual content; final approval of the version to be published
YC	Substantial contributions to the conception or design of the work; or the acquisition, analysis or interpretation of data for the work; drafting the work or revising it critically for important intellectual content; final approval of the version to be published
WP	Substantial contributions to the conception or design of the work; or the acquisition, analysis or interpretation of data for the work; drafting the work or revising it critically for important intellectual content; final approval of the version to be published
